# The value of repetitive transcranial magnetic stimulation combined with amisulpride enhancement olanzapine therapy for resistant treatment-refractory schizophrenia

**DOI:** 10.3389/fpsyt.2025.1715326

**Published:** 2026-01-05

**Authors:** Ou Yang, Liyan Yu, Yang Li, Miaozhen Zhou, Qianying Huang

**Affiliations:** 1 Department of Psychiatry, The Third People’s Hospital of Yongkang City, Yongkang City, Zhejiang, China; 2 Department of Geriatrics, Zone IV, Tongde Hospital of Zhejiang Province, Hangzhou, Zhejiang, China; 3 Mental Health Center, The Third People’s Hospital of Yongkang City, Yongkang City, Zhejiang, China

**Keywords:** repetitive transcranial magnetic stimulation, amisulpride enhancement therapy, olanzapine, resistant treatment-refractory, schizophrenia

## Abstract

**Objective:**

Repetitive transcranial magnetic stimulation (rTMS) is a widely used enhancement therapy for schizophrenia, but there are few controlled studies on the combination of rTMS and amisulpride enhancement therapy. The aim of this study is to explore the value of rTMS combined with amisulpride enhanced olanzapine therapy for resistant treatment-refractory schizophrenia (TRS) patients.

**Methods:**

We conducted a retrospective analysis based on records of TRS patients who received amisulpride enhanced olanzapine treatment at the Third People’s Hospital of Yongkang City from December 2022 to September 2023. Patients are divided into rTMS group and Non-rTMS group based on whether they receive combined rTMS treatment. We examined the patient’s Positive and Negative Symptom Rating Scale (PANSS) scores, Repeatable Battery for the Assessment of Neuropsychological Status (RBANS) scores, World Health Organization Quality of Life Questionnaire (WHOQOL-BREF), and incidence of adverse events.

**Results:**

After treatment, the scores of PANSS in both groups significantly decreased, and the rTMS group was lower than the Non-rTMS group (P<0.05); The scores of RBANS and WHOQOL-BREF in both groups significantly increased (P<0.05); Except for the attention sub item, all other sub scores in the rTMS group were higher than those in the Non-rTMS group (P<0.05); There was no significant difference in the total number of adverse events between the two groups (P>0.05).

**Conclusions:**

The combination of rTMS and amisulpride to enhance olanzapine treatment for TRS may offer potential benefits in symptom management, cognitive function, and quality of life.

**Core tip:**

rTMS is a widely used treatment method for schizophrenia. This study found that rTMS combined with amisulpride enhanced olanzapine treatment for TRS can significantly improve patients’ symptoms, cognitive function, and quality of life, with tolerability and safety.

## Introduction

Schizophrenia is a serious mental disorder characterized by hallucinations, delusions, disordered thinking, emotional apathy, and decreased willpower (1, 2). At present, clozapine is the only evidence-based antipsychotic drug used to treat TRS patients and is widely used in the treatment of schizophrenia (3). Clozapine can effectively block dopamine D2 receptors and 5-hydroxytryptamine (5-HT2A) receptors, thereby improving patients’ symptoms (3, 4). However, there are still 30% -50% of patients whose symptoms have not been effectively improved after treatment with clozapine, and even have recurrent or worsening conditions (3–6). For some patients, the use of clozapine has been excluded due to clinical contraindications, including discontinuation of clozapine due to adverse events. Therefore, alternative treatment is needed for those who do not want to start using clozapine due to concerns about side effects or the need for monitoring (4–6). The effectiveness of olanzapine in treating non TRS has been confirmed (7). In addition, studies has found that olanzapine is equally effective as clozapine in adult patients with TRS, schizophrenia like disorders, or schizoaffective disorders (7–9).

At present, there are many enhancement methods for TRS treatment, but each method has certain limitations (9–12). For example, when combined with amisulpride, which is a selective dopamine D2/D3 receptor antagonist, it has good therapeutic effects on both positive and negative symptoms of schizophrenia (9, 10). Zink et al. (11) also confirmed that amisulpride enhanced olanzapine therapy has important clinical significance in treating TRS to improve clinical symptoms and cognitive function, with tolerability and safety. However, combination therapy may increase the risk of drug interactions, leading to an increased incidence of adverse reactions and individual differences in treatment efficacy (12). Schmidt-Kraepelin et al. (12) found that the combination therapy of olanzapine and amisulpride did not effectively alleviate symptoms, but sexual dysfunction, weight and waist circumference increased significantly compared to the control group.

Repetitive transcranial magnetic stimulation (rTMS) is a non-invasive neuromodulation technique that has been proven effective in the treatment of psychiatric disorders. Zhu et al. (13) confirmed that rTMS can significantly improve cognitive function in schizophrenia patients with memory deficits. RTMS acts on the cerebral cortex through pulsed magnetic fields, altering the excitability of neurons and regulating brain neural function (13). The dual regulation of 5-HT and dopamine systems by amisulpride and olanzapine helps restore balance between these two neurotransmitter systems (9–11). However, current research on the combination of rTMS and amisulpride to enhance olanzapine in the treatment of TRS patients is still poor, and the effectiveness and safety of their combined use are not yet clear.

Therefore, this retrospective analysis aims to investigate the value of rTMS combined with amisulpride in enhancing olanzapine treatment for TRS. We hope to provide some reference for the clinical treatment of TRS.

## Materials and methods

This study is a retrospective observational comparison, based on the medical records of TRS patients who received treatment with amisulpride enhanced olanzapine at the Third People’s Hospital of Yongkang City from December 2022 to September 2023. The patients were divided into rTMS and non-rTMS groups based on whether they received rTMS therapy during hospitalization. This grouping reflected real-world clinical decisions made by treating psychiatrists, combined with patients’ or caregivers’ preferences. All patients received olanzapine combined with amisulpride as standard pharmacological treatment, while rTMS was offered as an optional augmentation strategy when clinicians believed it may enhance treatment response or when patients expressed a desire for additional non-pharmacological interventions. This design enabled the evaluation of the potential additive value of rTMS in TRS management.

### Inclusion criteria

- Meets the diagnostic criteria of TRS; Having received at least two first or second-generation antipsychotic drugs with different chemical structures (at least one of which is a second-generation antipsychotic drug), and each antipsychotic was administered for at least 6 consecutive weeks at a therapeutic dose equivalent to ≥300–600 mg/day of chlorpromazine. The definition of TRS used in this study follows the criteria proposed by Kane et al. (2), including inadequate response to two or more chemically distinct antipsychotics, sufficient dose and duration, and less than 20–30% reduction in Positive and Negative Symptom Scale (PANSS) positive symptoms (1);

- Age between 18 and 65 years old;
- Baseline PANSS score>60 points;
- Complete clinical data.

Exclusion criteria

- Patients with severe physical illnesses, such as heart, liver, and kidney dysfunction;
- Patients with organic brain lesions;
- Patients with drug abuse or alcohol dependence;
- Pregnant or lactating women.

Notably, none of the enrolled TRS patients had received clozapine prior to or during the study period. The reasons included: (1) clinical contraindications to clozapine use, such as metabolic syndrome or cardiac arrhythmias; (2) patient or family refusal due to concerns about side effects or frequent hematological monitoring; and (3) physician decision to delay clozapine initiation during early TRS stages while trialing other augmentation strategies. These real-world factors are consistent with current clinical practices and reflect a subgroup of TRS patients who remain clozapine-naïve.

Olanzapine (manufacturer: Jiangsu Enhua Pharmaceutical Co., Ltd.; specification: 5mg/tablet), taken orally, taken overnight before bedtime; Starting from 10 mg/d in the first week, 15 mg/d in the second week, and a maximum of 20 mg/d for the remaining six weeks.

Amisulpride (Manufacturer: Shenzhen Pangu; China; 50 mg/tablet) is used for treatment, orally, taken overnight before bedtime; Starting from 200 mg/d in the first week, 400 mg/d in the second week, and a maximum of 600mg/d for the remaining six weeks.

Repetitive transcranial magnetic stimulation (rTMS) was administered using the M-50 Ultimate stimulator (Shenzhen Yingzhi, China), equipped with a figure-of-eight coil. The stimulation site was the left dorsolateral prefrontal cortex (dlPFC), localized using the standard “5-cm rule,” defined as 5 cm anterior to the motor hotspot for the abductor pollicis brevis muscle. The stimulation frequency and intensity were individually adjusted for each patient within a predefined range of 10–20 Hz and 80–110% of the resting motor threshold (RMT), based on clinical response and tolerance. Each train lasted for 4 seconds, followed by a 56-second inter-train interval, with a total stimulation time of 20 minutes per session. Sessions were conducted once daily, 5 days per week, for a total duration of 8 weeks. All procedures were performed by the same group of rTMS-certified psychiatrists using the same device, ensuring procedural consistency and reproducibility.

Collect information: 1) Population characteristics, including age, gender, disease duration, educational level, family history, body mass index, and marital status. 2) The baseline and post-treatment PANSS scores include a positive symptom scale (7 items), a negative symptom scale (7 items), and a general psychopathological symptom scale (16 items), with each item scoring 1–7 points. The higher the score, the more severe the symptoms (11). 3) Baseline and post-treatment Repeatable Battery for the Assessment of Neuropsychological Status (RBANS) scores, including 12 items divided into five factor structures: attention, speech, visual breadth, immediate memory, and delayed memory; The higher the score, the better the patient’s cognitive function. 3) The baseline and post-treatment World Health Organization Quality of Life Questionnaire (WHOQOL-BREF) scores include four domains: social relationships, environment health, psychological well-being, and physical health, with a total score of 100 points for each dimension. The higher the score, the better the quality of life. 4) Adverse events include dizziness, dry mouth, constipation, insomnia, nausea and vomiting, extrapyramidal reactions, arrhythmia, etc.

Statistical analysis: Based on a statistical power of 0.80 and a two-tailed significance level of 0.05, the minimum required sample size per group was calculated to be 34 participants. Considering an anticipated dropout rate of less than 15%, we targeted a final sample size of 40 participants per group. All statistical analyses were performed using SPSS software (version 24.0, IBM Corp., Armonk, NY, USA). The Shapiro–Wilk test was used to assess the normality of quantitative data. Normally distributed variables were expressed as mean ± standard deviation (SD), and compared using the independent-samples t-test (between-group) or paired-samples t-test (within-group). Non-normally distributed data were presented as median and interquartile range (IQR), and compared using the Mann–Whitney U test or the Wilcoxon signed-rank test, as appropriate. Categorical variables were analyzed using the chi-square test or Fisher’s exact test. Statistical significance was defined as P < 0.05. Due to the retrospective design and inherent data limitations, no propensity score matching or regression-based confounding adjustment was performed. However, strict inclusion and exclusion criteria were applied, and baseline characteristics between groups were statistically compared, showing no significant differences (**Table 1**). Furthermore, as an exploratory analysis, we did not perform formal corrections for multiple comparisons across RBANS and WHOQOL-BREF subdomains (e.g., Bonferroni or false discovery rate methods), in order to preserve sensitivity. To enhance interpretability, we also reported effect sizes for key comparisons, including Cohen’s d for normally distributed continuous variables, r for non-normal distributions, and phi coefficients for categorical data. Future studies should incorporate appropriate statistical adjustments and larger samples to confirm these findings and reduce Type I error.

**Table 1:** Comparison of demographic characteristic variables between two groups.

Variables	rTMS group (n=64)	Non-rTMS group (n=69)	Cohen’s d/Phi/r	t/Z/*χ^2^*	P
Age, mean ± SD	39.8 ± 11.5	42.1 ± 10.7	-0.211	-1.216	0.228
Sex, n(%			0.000	0.000	1.000
Male	33 (51.6)	35 (50.7)			
Female	31 (48.4)	34 (49.3)			
Disease duration (years), M(IQR)	23 (16-26.5)	24 (16-31)	-0.119	-1.373	0.170
Educational level, median (IQR)	9 (7-9)	9 (6-9)	0.005	-0.059	0.954
Family history, n(%)			0.069	0.624	0.429
yes	19 (29.7)	26 (37.7)			
no	45 (70.3)	43 (62.3)			
BMI (kg/m²), mean ± SD	25.0 ± 2.8	24.2 ± 3.1	0.264	1.530	0.128
Marital status, n(%)			0.142	2.692	0.442
Unmarried	18 (28.1)	20 (29.0)			
Married	34 (53.1)	32 (46.4)			
Divorced	11 (17.2)	12 (17.4)			
Lose a spouse	1 (1.6)	5 (7.2)			

Data are presented as mean ± standard deviation, median (interquartile range), or number (percentage). Cohen’s d was calculated for normally distributed continuous variables, r for non-normally distributed continuous variables (Mann–Whitney U test), and Cramer’s V (or phi) for categorical variables.

## Results

In this retrospective observational comparison study, a total of 133 patients met the criteria. Among them, 53.4% (71/133) were male. Age range: 18–63 years old, with an average age of 41.0 ± 11.1 years old; There were 64 patients in the rTMS group and 69 patients in the Non-rTMS group. There were no statistically significant differences in demographic characteristics between the two groups (P > 0.05), and effect sizes (Cohen’s d, r, or φ) indicated small to negligible group differences (**Table 1**).

**Table 2** presents the PANSS scores of both groups before and after treatment, along with corresponding effect sizes. Prior to treatment, there were no statistically significant differences between the two groups in PANSS general psychopathology, positive symptoms, negative symptoms, or total scores (P > 0.05). After 8 weeks of treatment, both groups showed significant reductions in all PANSS domains (P < 0.05). The rTMS group exhibited significantly greater symptom improvement than the Non-rTMS group across all domains, with small to moderate effect sizes (Cohen’s d ranging from -0.258 to -0.545). These results are supported not only by statistical significance but also by the observed effect sizes, which reflect the magnitude of group differences.

**Table 2:** Comparison of PANSS scores between two groups before and after treatment.

Time	Variables	rTMS group (n=64)	Non-rTMS group (n=69)	Cohen’s d/r	Z/t	P
Before intervention	General psychopathology subscores, median (IQR)	42.5 (32-51.5)	43 (37-54)	-0.067	-0.777	0.438
	Positive symptom subscores, mean ± SD	25.1 ± 3.2	24.4 ± 3.6	0.259	1.497	0.137
	Negative symptom subscores, median (IQR)	23 (22-25)	25 (22-27)	-0.137	-1.578	0.113
	Total score, mean ± SD	90.5 ± 11.4	92.1 ± 12.4	-0.055	-0.635	0.527
After intervention	General psychopathology subscores, median (IQR)	20.5 (19-23.5)^#^	25 (22-29)^#^	-0.420	-4.848	<0.001
	Positive symptom subscores	15 (13-18)^#^	19 (16-20)^#^	-0.376	-4.337	<0.001
	Negative symptom subscores, median (IQR)	15 (12-16)^#^	16 (14-20)^#^	-0.258	-2.977	0.003
	Total score, median (IQR)	51.5 (48.5-54.5)^#^	61 (56-66)^#^	-0.545	-6.280	<0.001

Data are presented as mean ± standard deviation or median (interquartile range) as appropriate. Between-group comparisons were performed using independent-samples t-tests or Mann–Whitney U tests. Effect sizes are reported as Cohen’s d for normally distributed variables and r for non-normally distributed variables (Mann–Whitney U test). The symbol ^#^ indicates P<0.05 for within-group comparisons between before and after treatment.

**Table 3** shows the changes in RBANS subscale and total scores before and after treatment, along with effect sizes for between-group comparisons. Before treatment, there was no statistically significant difference in the immediate memory score, visuospatial/construction score, language score, attention score, delayed memory score, and total score between the two groups (P>0.05). After 8 weeks of treatment, both groups showed significant increases in immediate memory score, visuospatial/construction score, language score, attention score, delayed memory score, and total score compared to before treatment (P<0.05). Additionally, except for the attention domain, the rTMS group demonstrated significantly greater improvements in all other cognitive domains compared to the Non-rTMS group (P < 0.05), with effect sizes ranging from small to moderate (Cohen’s d = 0.211–0.631).

**Table 3:** Comparison of RBANS scores before and after treatment between two groups.

Time	Variables	rTMS group (n=64)	Non-rTMS group (n=69)	Cohen’s d/r	t/Z	P
Before intervention	Immediate memory, median (IQR)	63(56.2-69)	62(54-65)	0.136	1.565	0.118
	Visuospatial/construction, mean ± SD	67.2 ± 10.8	65.8 ± 9.2	0.147	0.843	0.401
	Language, median (IQR)	74.5(60.8-78)	73(67-78)	-0.028	-0.322	0.749
	Attention, mean ± SD	70.9 ± 9.3	69.3 ± 11.4	0.152	0.883	0.379
	Delayed memory, mean ± SD	60.8 ± 8.0	58.9 ± 8.1	0.246	1.417	0.159
	Total score, median (IQR)	63.5 (53-71.5)	62 (53-65)	0.080	0.923	0.357
After intervention	Immediate memory, mean ± SD	70.0 ± 9.5^#^	64.6 ± 7.5^#^	0.631	3.606	<0.001
	Visuospatial/construction, mean ± SD	72.4 ± 11.4^#^	67.5 ± 9.8^#^	0.462	2.644	0.009
	Language, mean ± SD	80.7 ± 14.7^#^	74.7 ± 10.0^#^	0.481	2.734	0.007
	Attention, mean ± SD	72.3 ± 8.4^#^	70.4 ± 9.8^#^	0.225	1.308	0.193
	Delayed memory, median (IQR)	68(59-85)^#^	63(54-66)^#^	0.317	3.652	<0.001
	Total score, median (IQR)	69.5 (59-81)^#^	65 (56-68)^#^	0.211	2.429	0.015

Data are presented as mean ± standard deviation or median (interquartile range) as appropriate. Between-group comparisons were performed using independent-samples t-tests or Mann–Whitney U tests. Effect sizes are reported as Cohen’s d for normally distributed variables and r for non-normally distributed variables (Mann–Whitney U test). The symbol ^#^ indicates P<0.05 for within-group comparisons between before and after treatment.

**Table 4** presents the changes in WHOQOL-BREF domain scores before and after treatment, including corresponding effect sizes. Before treatment, there was no statistically significant difference in the scores of the social relationship score, environmental score, psychological score, and physiological score between the two groups (P>0.05). After 8 weeks of treatment, both groups showed significant improvements in all WHOQOL-BREF domains (P < 0.05), with the rTMS group demonstrating significantly higher post-treatment scores than the Non-rTMS group. The between-group effect sizes ranged from small to moderate (Cohen’s d = 0.303–0.743).

**Table 4:** Comparison of WHOQOL-BREF scores between two groups before and after treatment​.

Time	Variables	rTMS group (n=64)	Non-rTMS group (n=69)	Cohen’s d/r	Z	P
Before intervention	Social relationships, median (IQR)	49 (45-52.5)	50 (45-54)	-0.079	-0.910	0.363
	Environment health, median (IQR)	49.5 (46.5-54)	50 (47-56)	-0.090	-1.036	0.300
	Psychological well-being, median (IQR)	46.5 (43-50)	45 (41-50)	0.108	1.247	0.212
	Physical health, median (IQR)	51.5 (47-55)	49 (45-56)	0.125	1.437	0.151
After intervention	Social relationships, median (IQR)	74 (69-79.5)^#^	69 (63-74)^#^	0.303	3.499	<0.001
	Environment health, mean ± SD	72.5 ± 5.9^#^	67.7 ± 6.9^#^	0.743	4.306	<0.001
	Psychological well-being, median (IQR)	74 (69-77.5)^#^	70 (64-75)^#^	0.333	3.837	<0.001
	Physical health	76 (71-79)^#^	70 (66-77)^#^	0.335	3.864	<0.001

Data are presented as mean ± standard deviation or median (interquartile range) as appropriate. Between-group comparisons were performed using independent-samples t-tests or Mann–Whitney U tests. Effect sizes are reported as Cohen’s d for normally distributed variables and r for non-normally distributed variables (Mann–Whitney U test). The symbol ^#^ indicates P<0.05 for within-group comparisons between before and after treatment.

**Table 5** summarizes the incidence and types of adverse events in both groups, along with effect sizes (phi coefficients). In the rTMS group, there were 12 cases of dizziness, 5 cases of dry mouth, 4 cases of constipation, 4 cases of insomnia, 5 cases of nausea and vomiting, 2 cases of extrapyramidal reactions, and 6 cases of arrhythmia. The total number of adverse events was 31, with an incidence rate of 48.4% (31/64), of which 7 people experienced at least two types of adverse events; In the Non-rTMS group, there were 7 cases of dizziness, 5 cases of dry mouth, 2 cases of constipation, 8 cases of insomnia, 8 cases of nausea and vomiting, 5 cases of extrapyramidal reactions, and 4 cases of arrhythmia. The total number of adverse events was 31, with an incidence rate of 44.9% (31/69), of which 8 cases experienced at least two types of adverse events. There was no statistically significant difference in the overall incidence of adverse events between the two groups (P > 0.05), and effect sizes (phi = 0.020–0.101) indicated small group differences.

**Table 5:** Occurrence of adverse events in two groups.

Adverse event	rTMS group (n=64)	Non-rTMS group (n=69)	Phi	*χ^2^*	P
Dizzy	12 (18.8)	7 (10.1)	0.101	1.367	0.242
Dry mouth	5 (7.8)	5 (7.2)	0.000	0.000	1.000
Constipation	4 (6.3)	2 (2.9)	0.044	0.263	0.608
Insomnia	4 (6.3)	8 (11.6)	0.067	0.596	0.440
Nausea and vomiting	5 (7.8)	8 (11.6)	0.038	0.195	0.659
Extrapyramidal syndrome	2 (3.1)	5 (7.2)	0.059	0.456	0.500
Arrhythmia	6 (9.4)	4 (5.8)	0.039	0.205	0.651
Total number of occurrences	31 (48.4)	31 (44.9)	0.020	0.054	0.817

All adverse events were mild to moderate in severity and self-limiting in duration. No patient discontinued treatment due to adverse effects. No rTMS-specific adverse events (e.g., headache, scalp discomfort, tinnitus, or seizures) were observed during the study period. Data are presented as number (percentage). Between-group differences in adverse event rates were analyzed using chi-square tests. Effect sizes are reported as phi coefficients for 2×2 contingency tables.

## Discussion

In this retrospective observational comparison study, we evaluated the efficacy and safety of combining repetitive transcranial magnetic stimulation (rTMS) with amisulpride to augment olanzapine treatment in patients with treatment-refractory schizophrenia (TRS). The results suggest that this combination may be associated with improvements in psychiatric symptoms, cognitive performance, and quality of life, without an increased risk of adverse events. This is consistent with the research results of Liu et al. (14) and Zhao et al. (15). This may be explained by insufficient D2 receptor occupancy in TRS patients under standard treatment, whereas amisulpride provides selective D2/D3 antagonism that enhances dopaminergic blockade (9–12, 14, 15). In addition, rTMS uses magnetic pulses to modulate cortical excitability, thereby regulating neural circuits implicated in psychiatric symptoms (13–15). It can be seen that combining rTMS enhanced therapy with drugs has broad prospects.

After eight weeks of treatment, the rTMS group exhibited significantly lower PANSS scores compared to the Non-rTMS group. This indicates that the combination of rTMS and amisulpride to enhance olanzapine treatment has more advantages in general symptoms, positive symptoms, and negative symptoms. This is consistent with the findings of Liu et al. (14) There are literature reports that when there is a poor response to olanzapine, relying solely on amisulpride to enhance treatment may not be able to fully correct the complex neurotransmitter imbalance in the patient’s brain (12, 16). However, rTMS regulates neurotransmitter levels and improves brain function through magnetic pulses, synergizing with amisulpride to more effectively improve patients’ psychiatric symptoms and reduce PANSS scores (13–15, 17). This is consistent with the findings of Lorentzen et al. (17).

The results of this study showed that in terms of RBANS scores, except for attention, the rTMS group had significantly higher scores in dimensions such as immediate memory, visual breadth, speech function, and delayed memory than the Non-rTMS group, indicating that combination therapy has a more significant improvement in patients’ cognitive function and can better help patients recover their memory and language expression abilities. The research results of Du et al. (18) indicate that rTMS improves visual memory and reduces negative symptoms of schizophrenia. Meanwhile, Xie et al. (19) confirmed that low-frequency rTMS can improve auditory hallucination speech function in schizophrenia. Mainly because rTMS stimulates the prefrontal cortex, which can increase dopamine release in this area and improve patients’ cognitive function, including memory and language related executive functions (18, 19). In terms of quality of life, the WHOQOL-BREF score in the rTMS group was higher than that in the Non-rTMS group. This indicates that after rTMS combined with amisulpride enhances olanzapine treatment to improve patients’ mental symptoms and cognitive function, their quality of life naturally improves. This may contribute to improved quality of life across physical, psychological, social, and environmental domains (18–20). In terms of safety, both groups reported the occurrence of adverse events, mainly dizziness, insomnia, and nausea and vomiting. But there was no significant difference in the total number of participants (48.4% in the rTMS group vs. 44.9% in the Non-rTMS group). This indicates that the combination of rTMS and amisulpride to enhance olanzapine is safe. This is consistent with previous studies (18–23).

Moreover, the effect size (Cohen’s d = 0.225) was small, supporting the statistical finding of a non-significant group difference in attention performance. Several explanations may account for this finding. First, the baseline attention scores were relatively high in both groups, suggesting a possible ceiling effect (24). Second, the left dorsolateral prefrontal cortex (dlPFC), which was targeted in rTMS, is more commonly associated with memory and executive function than attentional regulation (25). Third, the RBANS attention domain itself may have limited sensitivity in detecting short-term fluctuations in attentional performance among schizophrenia patients, as reported in previous studies (26). However, we recognize that the extent of improvement observed within 8 weeks may appear optimistic. These results may be influenced by patient selection factors (e.g., baseline impairment severity) or the natural variation of short-term treatment responsiveness. The lack of long-term follow-up and mechanistic biomarkers limits the ability to confirm sustained or causal benefits. Taken together, these findings should be interpreted with caution and considered exploratory.

In summary, this study examined the potential clinical value of combining rTMS with amisulpride to enhance olanzapine treatment in patients with TRS. The results suggest potential benefits in improving psychiatric symptoms, cognitive function, and quality of life, with an acceptable safety profile. The pathophysiology of TRS may be more complex than that of other psychiatric disorders that typically respond well to standard antipsychotic treatments, and it may even involve non-dopaminergic mechanisms (23, 27). Therefore, the combination of rTMS and pharmacological augmentation using amisulpride may offer a promising therapeutic strategy in such cases. However, the neurobiological mechanisms underlying this combined approach remain speculative. As this was a retrospective clinical study, no neuroimaging, electrophysiological, or neurotransmitter data were collected. Future prospective studies incorporating multimodal biomarkers—such as functional magnetic resonance imaging (fMRI), electroencephalography (EEG), or magnetic resonance spectroscopy (MRS)—are warranted to clarify the underlying mechanisms.

This study has several limitations. First, it is a single-center retrospective study with a relatively small sample size, which may limit the generalizability of the findings. Larger, multi-center prospective studies are warranted to validate these results. Second, although effect sizes were reported to enhance interpretability, we did not apply multiple comparison corrections (e.g., Bonferroni or FDR) across cognitive and quality of life subdomains, which may increase the risk of Type I error. This limitation is common in exploratory studies and should be addressed in future confirmatory research using appropriate statistical adjustment methods. Third, although no statistically significant differences were found in baseline characteristics between groups, the non-randomized design and grouping based on real-world treatment choices introduce potential selection bias and residual confounding. Therefore, the findings should be interpreted as associative rather than causal. Future studies employing randomized controlled or propensity score-matched designs are needed to strengthen causal inference. Fourth, the underlying mechanisms of rTMS combined with amisulpride augmentation of olanzapine remain unclear. As this was a clinical study without neuroimaging or biomarker data, further basic research is required to elucidate the neurobiological pathways involved. Finally, the long-term efficacy and safety of rTMS combined with pharmacological enhancement were not evaluated. Future investigations should assess the sustained effects of treatment, including long-term adverse drug reactions and the neuroregulatory impact of rTMS, and explore different stimulation parameters and dosage combinations to optimize treatment strategies.

## Conclusion

This study suggests that combining rTMS and amisulpride with olanzapine treatment may be associated with improvements in psychiatric symptoms, cognitive function, and quality of life in TRS patients, with acceptable safety. This suggests a potentially promising approach for TRS management and has important clinical application value. However, due to certain limitations of this study, more high-quality research is still needed in the future to further validate and improve this treatment plan, in order to better serve clinical practice and help more patients recover their health.

## Data Availability

The original contributions presented in the study are included in the article/supplementary material. Further inquiries can be directed to the corresponding author.
